# Effects of Soil Water and Nitrogen on Growth and Photosynthetic Response of Manchurian Ash (*Fraxinus mandshurica*) Seedlings in Northeastern China

**DOI:** 10.1371/journal.pone.0030754

**Published:** 2012-02-08

**Authors:** Miao Wang, Shuai Shi, Fei Lin, Zhanqing Hao, Ping Jiang, Guanhua Dai

**Affiliations:** Institute of Applied Ecology, Chinese Academy of Sciences, Shenyang, China; United States Department of Agriculture, Agricultural Research Service, United States of America

## Abstract

**Background:**

Soil water and nitrogen (N) are considered to be the main environmental factors limiting plant growth and photosynthetic capacity. However, less is known about the interactive effects of soil water and N on tree growth and photosynthetic response in the temperate ecosystem.

**Methods/Principal Findings:**

We applied N and water, alone and in combination, and investigated the combined effect of different water and N regimes on growth and photosynthetic responses of *Fraxinus mandshurica* seedlings. The seedlings were exposed to three water regimes including natural precipitation (CK), higher precipitation (HW) (CK +30%) and lower precipitation (LW) (CK −30%), and both with and without N addition for two growing seasons. We demonstrated that water and N supply led to a significant increase in the growth and biomass production of the seedlings. LW treatment significantly decreased biomass production and leaf N content, but they showed marked increases in N addition. N addition could enhance the photosynthetic capability under HW and CK conditions. Leaf chlorophyll content and the initial activity of Rubisco were dramatically increased by N addition regardless of soil water condition. The positive relationships were found between photosynthetic capacity, leaf N content, and SLA in response to water and N supply in the seedling. Rubisco expression was up-regulated by N addition with decreasing soil water content. Immunofluorescent staining showed that the labeling for Rubisco was relatively low in leaves of the seedlings under LW condition. The accumulation of Rubisco was increased in leaf tissues of LW by N addition.

**Conclusions/Significance:**

Our study has presented better understanding of the interactions between soil water and N on the growth and photosynthetic response in *F. mandschurica* seedlings, which may provide novel insights on the potential responses of the forest ecosystem to climate change associated with increasing N deposition.

## Introduction

Human activities such as fossil fuel burning, forest disturbance, and land conversion have globally elevated the atmospheric concentration of carbon dioxide (CO_2_) and atmospheric deposition of nitrogen (N) [1]. The atmospheric N depositions are altering the availability of this limiting nutrient in many terrestrial ecosystems [2]. Elevated N availability can affect plant growth, biodiversity, and ecosystem functioning [3], [4]. Soil N availability has the potential to alter plant physiology in terrestrial ecosystems [5], [6]. Increases in atmospheric N deposition can affect the amount of N available to plants which influence the growth and survival of the seedlings [7]. Photosynthesis may be altered in responses to elevated N availability [8]. Increased N availability results in increased photosynthesis and growth in northern hardwood trees [9]. N additions increase leaf N concentrations accompanied by higher net photosynthetic rates in Douglas-fir [10], poplar [11], pond pine and red maple [12]. Maximum photosynthetic capacity is strongly regulated by leaf N concentration [13]. It is showed that there is a significant and positive correlation between photosynthetic capacity and leaf N content [14]–[16]. Increases in N availability have been shown to correspond with increased leaf chlorophyll content [11], [17], Rubisco (Ribulose-1, 5-bisphosphate carboxylase/oxgenase) [10]. N addition enhances tolerance of plants to abiotic stresses such as water deficits, salt and high temperatures [18]–[20]. Despite the potential importance of N deposition in plant, there is still limited knowledge regarding the relationship between N application, photosynthesis and growth in temperate forest ecosystems.

Soil water content is the primary limitation in photosynthetic processes in plants. Water availability influences leaf phenology [21] and photosynthetic rate [22]. It is well known that one of the primary physiological consequences of drought is photosynthesis inhibition [23], [24]. Inhibition of photosynthesis under drought has been attributed mainly to stomatal closure, reduced mesophyll conductance, and inhibition of Rubisco activity [25]–[29]. The major effects of water deficit on plant function include decreased shoot growth due to decreased leaf biomass and leaf area allocation, and increased leaf N content [30].

Physiological responses of plants to either water deficit or nitrogen addition have been documented [31]. Soil N availability can be affected by soil water availability via several microbial-mediated pathways, such as litter decomposition [32] and N mineralization [33]. Appropriate N supply is recommended to improve photosynthetic efficiency under water stress [15]. However the interactions between these two factors on plant physiological responses have received relatively little attention [34]. The overall effect of N addition and water changes on trees remains still unclear.

Rubisco is a kind of special enzymes that catalyzes the initial fixation reaction of photosynthesis [35]. Rubisco is mainly located in the chloroplasts of the bundle sheath cells in the leaves of higher plants. The large subunits of Rubisco play an important role in photosynthesis for CO_2_ assimilating [35]. Some evidences suggest that Rubisco functions increasingly as a storage protein in addition to its catalytic functions with increasing N_area_ [36]. The response of Rubisco to N supply in trees remains equivocal. The results show greater concentration of Rubisco in seedling foliage at high rates of N supply [37], whereas another study found no effect of fertiliser application on Rubisco concentration, Rubisco activity or photosynthesis in 25- to 30-year-old trees [38]. Less is known about the relationships between photosynthetic capacity, leaf N content, and the expression and activity of Rubisco in response to N and water in the seedling.

*Fraxinus mandschurica* is the most economically important forest tree species and primarily distributed in the temperate forests of northern hemisphere. However, forest decline of *F. mandschurica* have been recently observed in forest areas in the northeast in China due to logging and hunting. Protection and restoration of this ecologically important deciduous tree in temperate forest regions is crucial. Little information is available regarding the effects of N depositions and water availability on the growth and photosynthetic responses of *F. mandschurica* seedlings. Therefore, in the present study we applied N and water, alone and in combination, and investigated the interactive effects of N addition and soil water on the growth and physiological function of *F. mandschurica* seedlings. We specifically aimed to examine potential impacts of increased soil N and water availability and their interaction on whole-plant growth, biomass allocation, photosynthetic gas exchange, specific leaf area, leaf N content and photosynthetic pigment content in the seedlings. We also studied the changes in the expression and activity of Rubisco to clarify how N addition and water treatment affect photosynthetic functions of the seedlings. Better understanding of the interactions between soil water and N on trees may provide critical insights on the potential responses of the forest ecosystem to climate change associated with increasing atmospheric N deposition.

## Materials and Methods

### Study site

This study was carried out in the Changbai Mountain Natural Reserve in northeastern China (42°24′09″N, 128°05′45″E). The area is situated in the temperate continental climatic zone. Altitude above sea level of the study site is 738 m. Mean annual temperature is 3.6°C with monthly mean temperatures of −15.6°C in January and 19.7°C in July, respectively. Mean annual precipitation is 695 mm. The period of snow cover is from November to April, with a maximum depth of about 30 cm. Most precipitation in this area occurs from June to September (480–500 mm) [39]. The soil is classified as dark brown forest soil (Calcis-orthic Aridisol in the US Soil Taxonomy classification) with pH of 5.85, and with the top 30 cm containing an average of 156.6 g kg^−1^ organic carbon and 7.17 g kg^−1^ total N. The temperate broad-leaved Korean pine (*Pinus koraiensis*) mixed forest in the study area is dominated by *Pinus koraiensis* Sieb. et Zucc., *Fraxinus mandschurica* Rupr., *Quercus mongolica* Fisch. ex Ledeb. and *Tilia amurensis* Rupr.

### Experimental design

**Figure 1:**
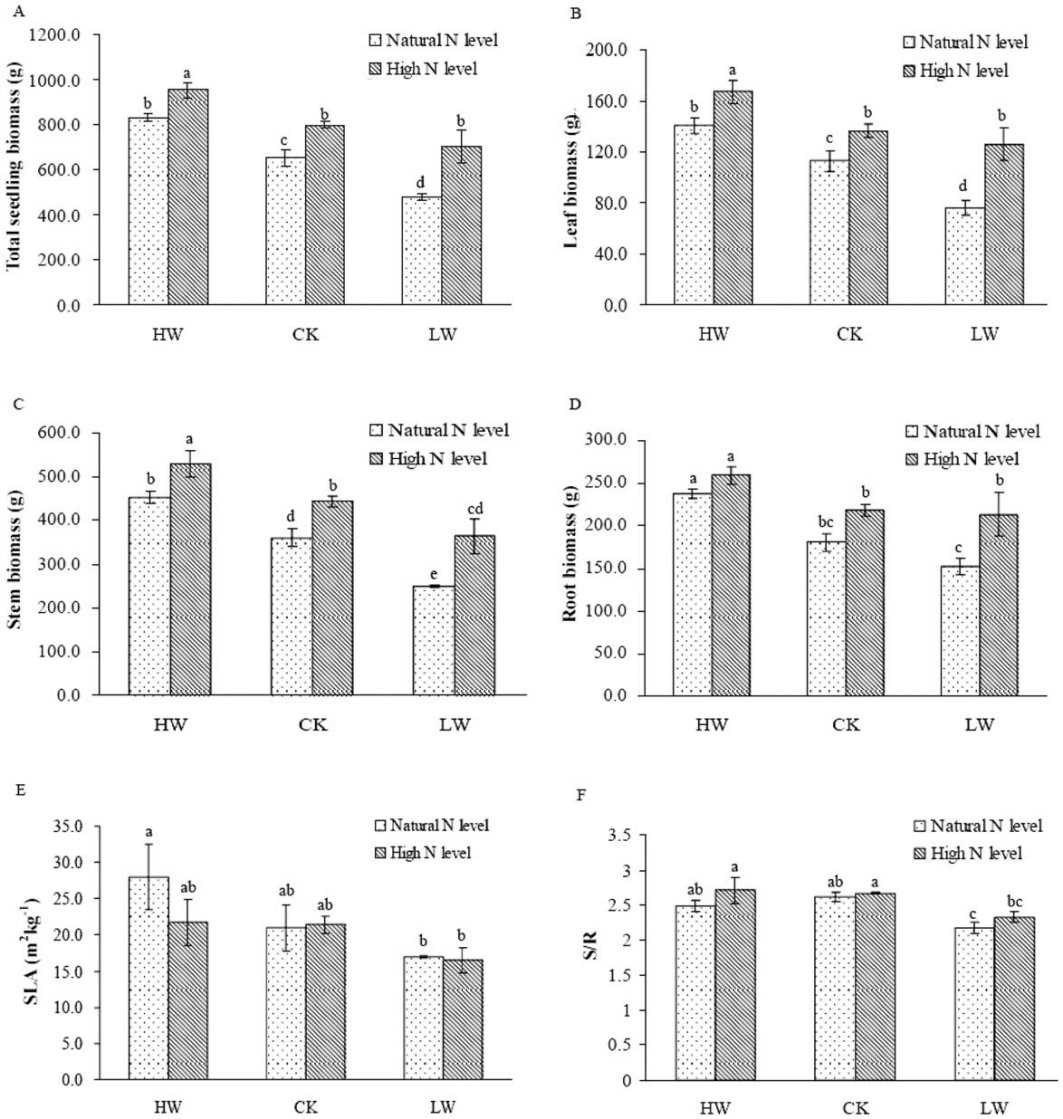
Total seedling, leaf, root and stem biomass (panels A–D) and SLA and S/R (panel E and F) under high-water (HW), (CK), and low-water (LW) conditions in combinations with natural (dotted) or high N-supply level (hatched). Bars represent means of 6 replications ± standard deviation. Values accompanied by different letters differ significantly at p = 0.05. Abbreviations: SLA, specific leaf area; S/R, the ratio of the stem and root biomass.

The experiment was conducted in openings within a mature broad-leaved Korean pine (*P. koraiensis*) mixed forest. A paired, nested design was used with precipitation as the primary factor and N addition the secondary one. The experiment involved three pairs of 21.6×1.6 m plots. N was added to one plot in each pair (+N), while the other plot in that pair contained no addition of N, but only that resulting from naturally occurring addition (CK). Each pair was also subjected to one of three water (precipitation) regimes: a) naturally occurring precipitation (CK); b) precipitation deduction (LW), in which 33% of naturally occurring precipitation was removed and diverted to c) precipitation enhancement plot (HW). These three regimes were applied to the three pairs of plots, yielding an overall experimental design as depicted in Figure 1. Each of the 6 plots was divided into nine 2.4×1.6 m subplots that served as replicates, yielding a total of 54 subplots in the experiment.

On 5 May 2006, two-year-old seedlings of *F. mandshuric*a were planted individually in the 54 subplots that served as locations for experiment replications. Precipitation was manipulated by means of troughs (0.16×1.6 m) suspended above the dry plots such that about 33% of the precipitation was trapped and passively transferred by gravity to polyvinylchloride piping and then across an ambient plot to a wet plot. In order to allow sunlight in, these flumes were made of transparent plastic board. The flumes inclined to the ground level at the angle of 15° with the highest and lowest points 1.43 m and 1 m above the ground, respectively. Flumes were spaced 40 cm apart. Soil water treatments began on 15 May 2006. To reduce nutrient heterogeneity, the original soil was excavated to a depth of 0.3 m and replaced with soil collected from the floor of a mature broad-leaved Korean pine (*P. koraiensis*) mixed forest. The soil was passed through a 4 mm sieve after collection.

Two N levels were control (CK) without N addition and N addition (+N) 10 g N m^−2^ yr^−1^ experimental input. The latter was applied by use of a backpack sprayer. Ammonium nitrate was applied twice per year on 15 May and 15 July in 2006, 2007 and 2008 as two equal applications (5 g N m^−2^, i.e. 54.86 g NH_4_NO_3_) over the entire year. During each application, fertilizer was weighed and mixed with 20 L of water. For each of the water treatments, soil volumetric water content (v/v) was periodically measured in the 0–30 cm depth range with a portable time domain reflect meter (TDR 100 Campbell, USA). Whole seedling dry mass, tree height and stem base diameter at the beginning of the experiment were 8.64±0.49 g, 26.35±0.80 cm and 8.33±0.20 mm, respectively. For assessment of water × N effects on the physiological/morphological characteristics of *F. mandshurica* seedlings, all seedlings were grown under the same conditions with the exception of variations in soil water and N levels.

### Growth parameters

In late September 2008, 36 randomly selected seedlings were harvested (n = 6 per replicate) to determine final shoot height, root collar diameter, and stem, leaf, and root biomass. Roots were separated from shoots by severing the seedling at the root collar, and were then carefully washed clean of growth media. The shoots were divided into leaf and stem components. Seedling fractions were oven-dried separately for at least 72 h at 80°C and the dry mass of each fraction was determined. Specific leaf area (SLA cm^2^ g^−1^) was measured on six seedlings for each treatment, using a LI-3000 leaf area meter (Li-Cor, Lincoln, NE).

### Gas exchange parameters

To characterize water- and N-induced shifts in carbon acquisition, instantaneous gas exchanges on fully expanded, exposed current-year leaves were measured under controlled optimal conditions using an open-mode portable photosynthesis system (LI-6400, Li-Cor, Lincoln, NE). For each treatment, three to four leaves of three individuals per replicate were randomly selected for sampling. For each seedling a series of five measurements per leaf was averaged (after the system had achieved a predetermined stability point), and the mean value of three individuals was used as the replicate for statistical analysis. *P*N-PAR response curves were measured at 1800, 1500, 1200, 1000, 800, 500, 200, 100, 50, 20, and 0 µmol m^−2^ s^−1^ of PAR under uniform conditions (25°C, 360±10 µmol (CO_2_) mol^−1^, and 65–75% RH at 9:00–11:30 on two sunny days. Maximum net photosynthetic rate (*A_max_*) and saturation irradiance were estimated according to Ellsworth (2000) [40]. All the measurements were recorded 5 times. In addition, water use efficiency was calculated using instantaneous measurements. Instantaneous water use efficiency (WUE_i_) was calculated and defined as *A_max_*/*E*, which *A_max_* is the light-saturated net CO_2_ assimilation rate and *E* is transpiration rate. All of the measurements were taken between 9:00 am to 11:30 pm on two fully sunny days (July 17–21, 2008) under natural conditions.

### Determination of N concentration per unit leaf area

On 15 June, 17 July and 20 August 2008, two or three nonshaded leaves per seedling were harvested and washed with deionized water. The area of the fresh leaves was measured after petiole removal, with an area meter (LI-3000A; Li-Cor). The harvested leaves were dried at 70°C during 48 h and ground for analysis. The specific leaf area (SLA) was determined as the ratio of leaf area to leaf dry mass, 20 leaves were collected and transported to the laboratory in refrigerated bags to avoid weight loss by respiration in each treatment. The leaf area was measured, after petiole removal, with an area meter (LI-3000A; Li-Cor). The dried leaves were ground to fine powder with a vibrating sample mill (MM-400 Retsch, Haan, Germany). The concentration of N in the powder was determined with a CHN analyzer (Vario EL; Elementar, Hanau, Germany). The N concentration per unit leaf area (N_area_) was determined as the ratio of N concentration to SLA of the leaves. Photosynthetic N-use efficiency (*PNUE*) was determined as the ratio of A360 to N_area_. [41].

### Photosynthesis pigment content

The fully expanded leaves from each seedling were collected, placed between layers of ice in a thermal insulated box, and taken to the laboratory of the National Research Station of Changbai Mountain forest ecology where they were analyzed immediately. The leaf disks (1 cm^2^) were taken and homogenized in chilled 80% (v/v) acetone, and the homogenates were centrifuged at 10000 g for 10 min at 4°C in the dark. The supernatant was used for determining pigment contents. The absorbance of the supernatant was recorded at 470, 646, and 663 nm. The amounts of chlorophyll a, b, and total chlorophyll were calculated as described by Inskeep and Bloom [42]. Total carotenoids were calculated as described by Arnon [43]. All the spectrophotometric assays were conducted using a UV-1601 spectrophotometer (Shimadzu, Japan).

### Measurement of Rubisco activity and activation state

On 18th August 2008, three non-shaded first-flush leaves per seedling were harvested from 9:00 to 11:30 am. The harvested leaves were washed with deionized water. Leaf samples (0.1 g) were frozen in liquid nitrogen until the measurements of activity and concentration of Rubisco. The stored leaf samples were homogenized to a fine powder in liquid nitrogen with a mortar and pestle. Subsequently, Rubisco was extracted by grinding the fine powder in a 1.0 ml extraction buffer containing 50 mM HEPES-KOH (pH 8.0), 10 mmol/L MgCl_2_, 0.5 mmol/L EDTA, 1% (w/v) polyvinylpolypyrrolidone. The crude homogenate was centrifuged at 16000 *g* for 15 min. The supernatant of the sample was used in the assay of activity of Rubisco. The activity of Rubisco was determined spectrophotometrically by measuring the disappearance rate of NADH [44]. To determine the initial activity of Rubisco, immediately after combining the desalted sample solution (100 µL with assay solution containing 50 mM HEPES-KOH (pH 8.0), 10 mM NaHCO_3_,1.5 mM NADH, 5 mM ATP, 1 mM EDTA, 20 mM MgCl_2_, 2.5 mM DTT, 5 mM phosphocreatin, 10 units per ml of phosphoglyceric kinase, 10 units per ml of glyceraldehyde-3-phosphate dehydrogenase and 20 units per ml of phosphocreatine kinase at final concentration, the reaction was started by adding 60 µL of 10 mM RuDP. The change in the absorption of the activation state of Rubisco was calculated as the ratio of initial activity to total activity of this enzyme.

### Western blotting

Leaf samples were ground in liquid N_2_ with mortar and pestle. Total proteins were extracted with a buffer containing 50 mM phosphate buffer solution (pH 7.5), 2% b-mercaptoethanol, 100 mM EDTA, 1% PVPP (w/v), and 1% Triton X-100 (v/v). After 15 min centrifugation (4°C, 15000 *g*), the upper phase was transferred to a new centrifuge tube. Two volumes of TRIS saturated phenol (pH 8.0) were added and then the mixture was further vortexed for 30 min. Proteins were precipitated by adding 5 vols of ammonium sulphate-saturated methanol, and incubated at −20°C for at least 4 h. After centrifugation as described above, the protein pellets were re-suspended and rinsed with ice-cold methanol followed by washing with ice-cold acetone twice, and spun down at 15000 *g* for 10 min at 4°C after each washing. Finally the washed pellets were air-dried and recovered in the lysis buffer containing 62.5 mM TRIS-HCl (pH 6.8), 2% SDS (v/v), 10% glycerol (v/v), and 2% β-mercaptoethanol (v/v). Protein concentrations were quantified using the Bradford assay [45].

For Western-blot analysis, an aliquot of the proteins (20 µg) was separated by SDS-PAGE using 12% (w/v) acrylamide gels according to the method of Laemmli (1970) and electrophoretically transferred to nitrocellulose membranes (Millipore, Saint-Quentin, France). The protein blot was probed with a primary antibody of the Rubisco large subunit (AS03037-200, Agrisera, Sweden) at a dilution of 1∶5000 for 4 h at room temperature with agitation. The blot was washed three times in phosphate buffered saline with Tween-20 solution containing 50 mM TRIS-HCl (pH 8.0), 150 mM NaCl, 0.05% Tween-20 (v/v), and followed by incubation with the secondary antibody (anti-rabbit IgG horseradish peroxidase conjugated, Abcam, UK, 1∶5000 dilution) for 2 h at room temperature. The blots were finally washed as above and developed with SuperSigmal West Pico Chemiluminescent Substrate (Pierce, USA) according to the manufacturer's instructions. Images of the blots were obtained using a CCD imager (FluorSMax, Bio-Rad, USA). The QuantityOne software (Bio-Rad, Hercules, CA, USA) was used to determine the optical density.

### Immunolocalization

Leaf sections were embedded in OCT compound (Sakura Finetek CA, USA) and sections were cut using a microtome and adhered to a poly-lysine coated slide. Sections were then fixed in 3% paraformaldehyde. After being rinsed with phosphate-buffered saline (PBS; 150 mM NaCl, 5 mM KCl, 0.8 mM KH_2_PO_4_, 3.2 mM Na_2_HPO_4_, pH 7.3), tissue sections were blocked with 1% bovine serum in PBS. Samples were washed extensively in PBS and then incubated at 4°C overnight with the polyclonal primary rabbit anti-Rubisco (1∶2000) in 0.5% BSA in PBS. After two washings in PBS, samples were incubated with anti-rabbit secondary antibody conjugated to Alexa 635 (1∶500) (Molecular Probes, Eugene, OR) for 30 min. Nuclei were stained with DAPI (4′, 6′-diamidino-2-phenylindole) (Molecular Probes, Eugene, OR). Slides were viewed with a Leica TCS SP2 confocal scanning microscope (Leica Microsystems, Heidelberg GmbH, Mannheim, Germany). Images were composed and analysed using Adobe PhotoShop 8.0.

### Statistical analyses

All statistical analyses were performed using SPSS 10.0 (SPSS, Chicago, Il, USA). Effects of soil water, N addition interaction between soil water and N addition were analyzed using a two way ANOVA (*p*<0.05). Differences between the means among soil water or N addition treatments were compared using Duncan's multiple range tests at <0.05 probability levels. For relationships of photosynthesis rate with leaf N content and SLA analysis was also performed (*p*<0.05). All the means involved in the interaction were compared. All data were presented as mean ± SD.

## Results

### Growth of the seedlings

Water and N addition had significant effects on seedling growth. A significant interactive effect of N addition and water treatment on plant height and root collar diameter of *F. mandshurica* seedlings was found as described in Table 1. N addition stimulated a significant increase in the height in HW treatment and root collar diameter of the seedlings in different water treatments (*p*<0.05). These two parameters markedly decreased under LW condition compared with that of the seedlings in CK, whereas N addition ameliorated the reduction. Soil water and N addition had advantaged effects in height growth and root collar diameter. N addition, water regimes and their interaction significantly influenced total seedling biomass, aboveground biomass (Fig. 1A–C). The significant changes were detected in total seedling biomass and above-ground biomass by N addition under HW and CK conditions (*p*<0.05). Even under LW treatment, N addition also led to a significant increase in total seedling biomass, leaf biomass and stem biomass (*p*<0.05). N addition increased root biomass especially in LW treatment. The ratio of the stem and root biomass (S/R) showed a significant decrease in LW treatment compared with that in CK (Fig. 1F). But no obvious changes were detected in S/R ratio by N addition, suggesting that N addition might not affect biomass allocation of the seedlings.

**Table 1:** The effects of N addition on plant height and root collar diameter of *F. mandshurica* seedlings grown in three different soil water regimes and two N treatment leaves.

Growth characteristics	Treatment					
	Natural N level			High N level		
	HW	CK	LW	HW	CK	LW
Plant height (m)	2.65±0.05^b^	2.67±0.06^b^	2.35±0.09^c^	2.85±0.03^a^	2.63±0.08^b^	2.52±0.11^b^
Root collar diameter (mm)	30.63±0.24^b^	29.26±0.87^b^	25.89±0.89^c^	35.76±0.29^a^	33.56±0.92^a^	29.02±1.30^b^

Values are mean ±SD of six replicates. And the same letter in the same row are not significantly different between treatments at the *p*<0.05 level.

### Gas exchange

**Figure 2:**
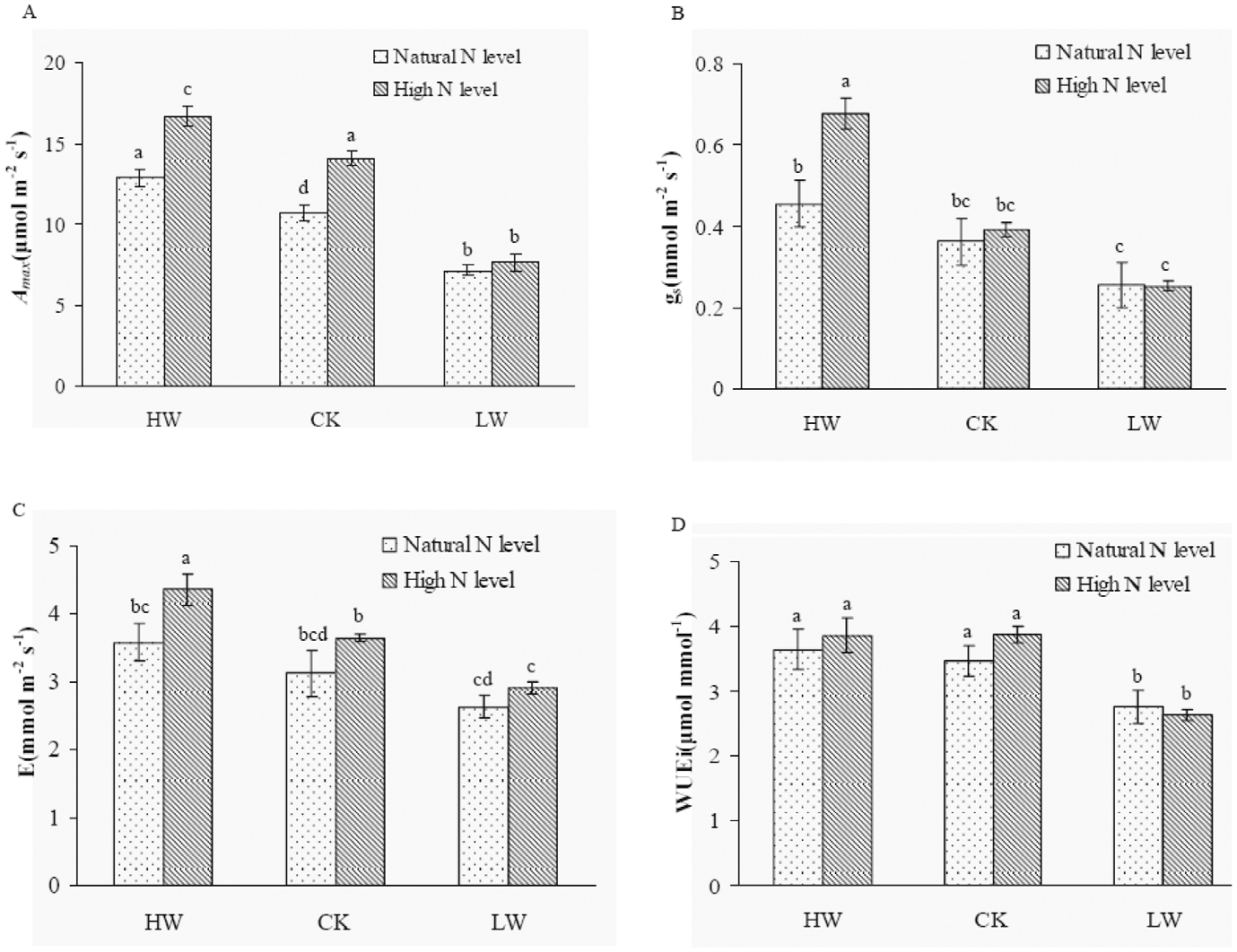
Effects of nitrogen addition and water regime on gas exchange. Parameters include: A. *A_max_*, B. *g*_s_, C. WUEi, D. E. Each column represents means ± SD (n = 6). Different letters indicate significant differences among treatments at p = 0.05. Abbreviations: *A_max_*: maximum photosynthetic rate; *g*_s_, stomatal conductance; WUEi intrinsic water use efficiency; *E*, transpiration.

The responses of *A_max_*, stomatal conductance (*g_s_*), WUEi and *E* in leaves of F. mandshurica seedlings to N and water were shown in Fig. 2. Leaf *A_max_* significantly increased by N addition under HW and CK, whereas this was not affected by N apply in LW treatment (Fig. 2A). The HW treatment alone induced a slight increase in *g*_s_, while the combination of N and HW treatment led to a dramatic enhancement of *g*_s_ (Fig. 2B). Similar to the responses of *g*_s_, leaf transpiration rate was significantly enhanced by N addition under HW (Fig. 2C). However, N addition didn't change the transpiration rate under CK and LW conditions. However, there was significant change in *g*_s_ in response to N addition under CK and LW conditions. A significant decrease in WUEi was found in LW treatment, whereas N addition didn't affect WUEi (Fig. 2D). A slight increase in WUEi but no significant difference was found by N addition under HW and CK conditions.

### Leaf N content and photosynthetic nitrogen-use efficiency (PNUE)

**Figure 3:**
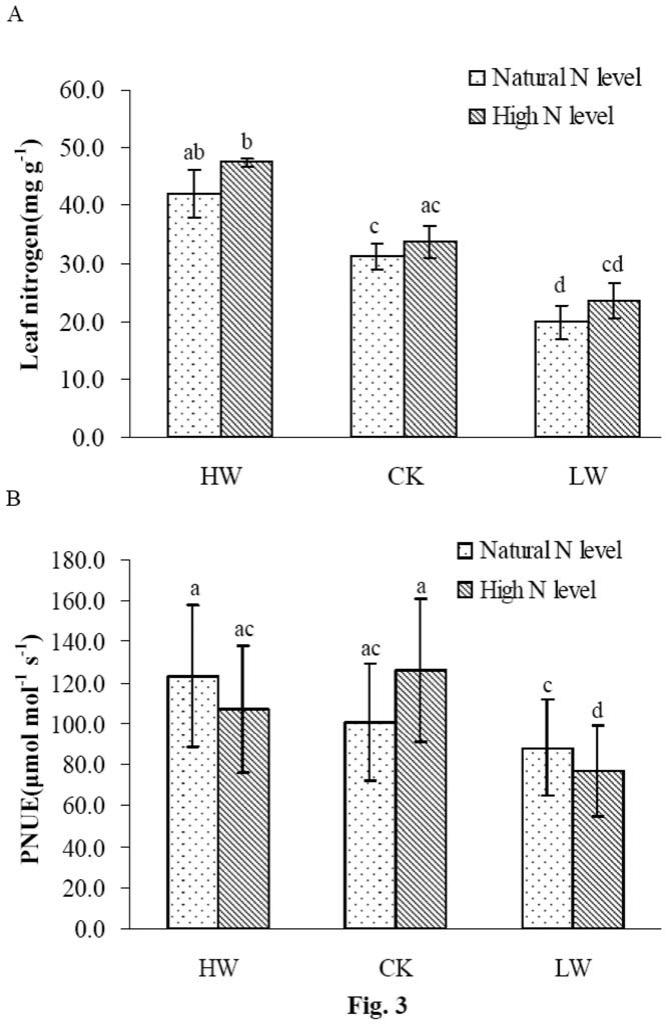
Effects of nitrogen addition and water regimes on leaf nitrogen (panel A) and photosynthetic nitrogen-use efficiency (PNUE) (panel B). Each column represents mean ± SD (n = 6). Mean values sharing the same letter are not significantly different among treatments (p>0.05).

**Figure 4:**
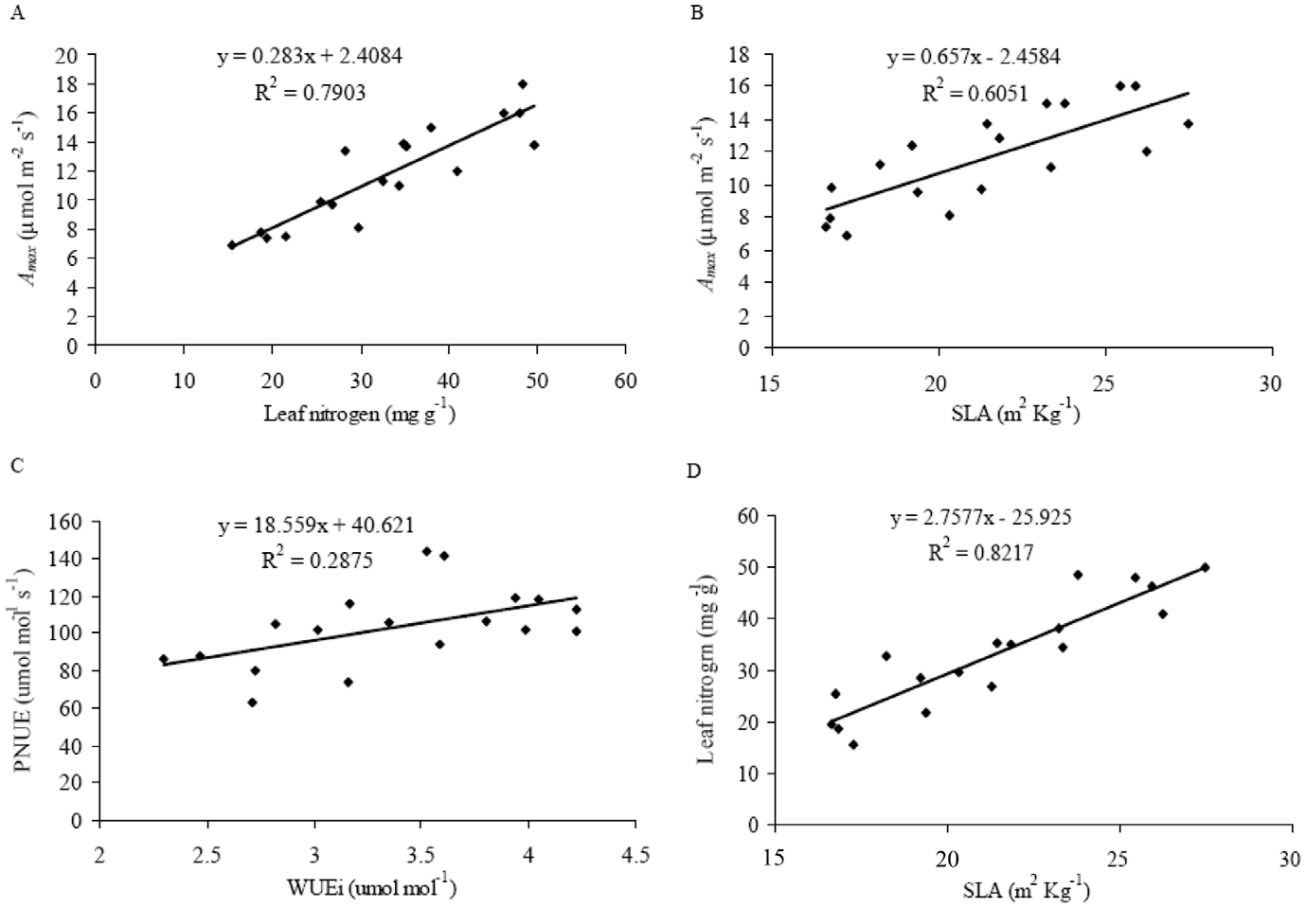
Correlations between *A_max_ vs.* leaf N (panel A), *A_max_ vs. SLA (panel B), PNUE* *vs. WUEi (panel C) and leaf N* *vs. SLA (panel D).* Data points are means of data from all the different water regimes and N treatments.

In order to examine possible relationships between photosynthesis and N availability in different soil water and N addition conditions, the effects of soil water and/or N addition on leaf N content and photosynthetic nitrogen-use efficiency (PNUE) were determined in the leaves of seedlings. There were significant differences in leaf N content between soil water treatments (Fig. 3A). HW treatment induced a significant increase in leaf N content, and this increase was further enhanced by N addition. Similarly, N addition also led to an elevation in leaf N content under CK and LW conditions (Fig. 3A). These results showed an interactive effect of soil N and water on the leaf N content in *F. mandshurica* seedlings. Leaf N content displayed significant positive correlation with *A_max_* (r^2^ = 0.79, *p*<0.05, Fig. 4A) and was positive with SLA (r^2^ = 0.60, *p*<0.05, Fig. 4C). SLA was also positively correlated with *A_max_* (r^2^ = 0.82, *p*<0.05, Fig. 4B). No significant variations in PNUE were found under different soil moisture (Fig. 3B). N addition resulted in a marked decrease for PNUE in LW treatment (Fig. 3B).

### Leaf chlorophyll content

**Figure 5:**
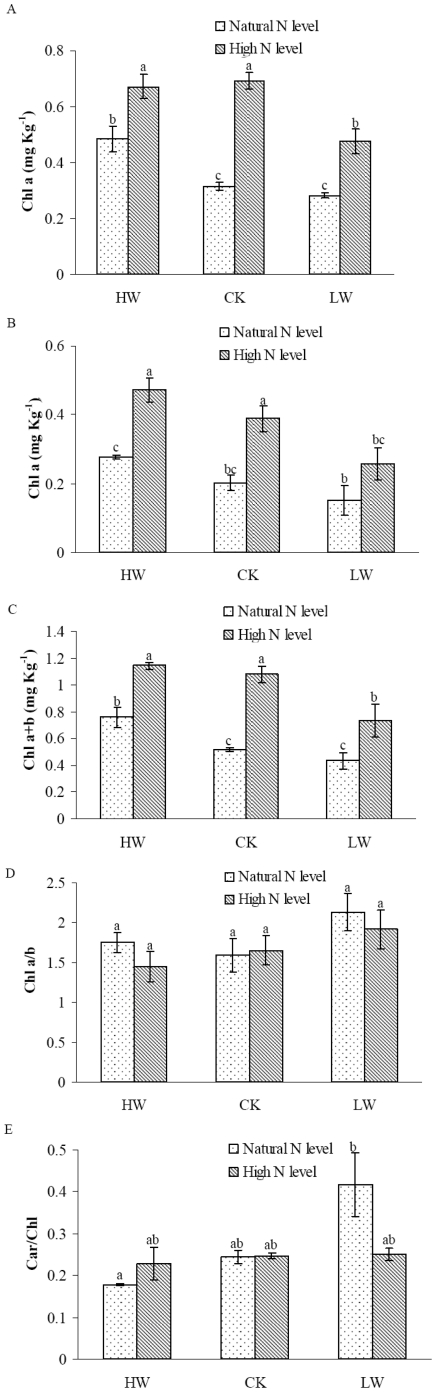
Impacts of nitrogen addition and water regimes on chlorophyll. Parameters include: A. Chl a, B. Chl b, C. Chl (a+b), D. Chl a/b, E. Car/Chl. Each column represents mean ± SD (n = 6). Mean values sharing the same letter are not significantly different among treatments (*p*>0.05). Abbreviations: Chl, chlorophyll; Car, carotinoid.

To examine how N addition and soil water affect photosynthetic capacity, we determined the response of leaf chlorophyll contents under N apply and different soil moisture conditions. The concentrations of leaf chlorophyll including total Chl (a+b), Chl *a* and Chl *b* were significantly influenced by N supply and water treatments in *F. mandshurica* (*p*<0.05) (Fig. 5A–C). N supply had significant positive effects on Chl a, Chl b and Chl (a+b) regardless of soil water contents. Chl a/b ratios were not significantly different in N addition or water treatment (Fig. 5D). In addition, the ratio of Car/Chl was dramatically increased under LW treatment (Fig. 5E). However, N addition enhanced the synthesis of chlorophyll, which leading to a recovery in the ratio of Car/Chl under LW condition (Fig. 5E).

### Rubisco activity

**Figure 6:**
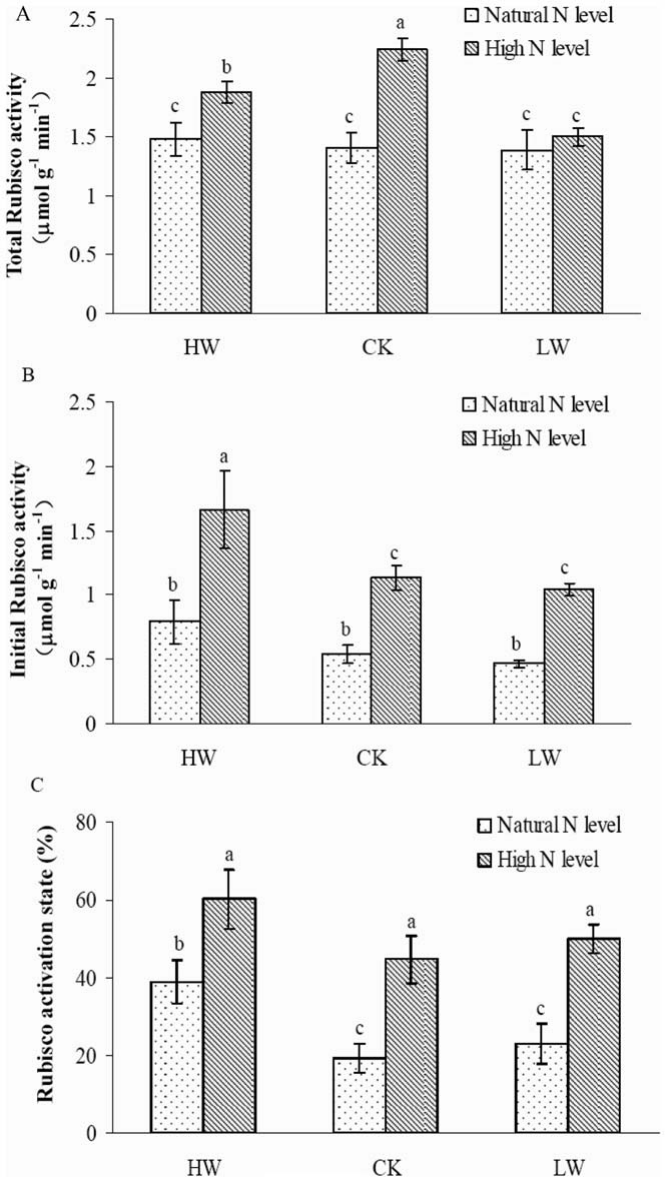
Total and initial Rubisco activity and Rubisco activation state (panels A–C) under HW, CK, and LW conditions in combinations with natural (dotted) or high N-supply level (hatched). Bars represent means of 6 replications ± standard deviation. Values accompanied by different letters differ significantly at p = 0.05.

Rubisco catalyzes CO_2_ assimilation and is a major limited factor in leaf photosynthetic responses of plants. We determined the effects of N addition and water regimes on the total activity and initial activity of Rubisco, as well as Rubisco activation state (Fig. 6). Water treatments had no significant effects on total Rubisco activity at natural N level (Fig. 6A). Total Rubisco activity was significantly increased at high N level in HW and CK. In addition, the initial activity of Rubisco was not affected by water regimes at natural N level and it was dramatically increased at high N level in all water treatments (Fig. 6B). Rubisco activation state was significantly increased by N supply (Fig. 6C).

### Expression of Rubisco

**Figure 7:**
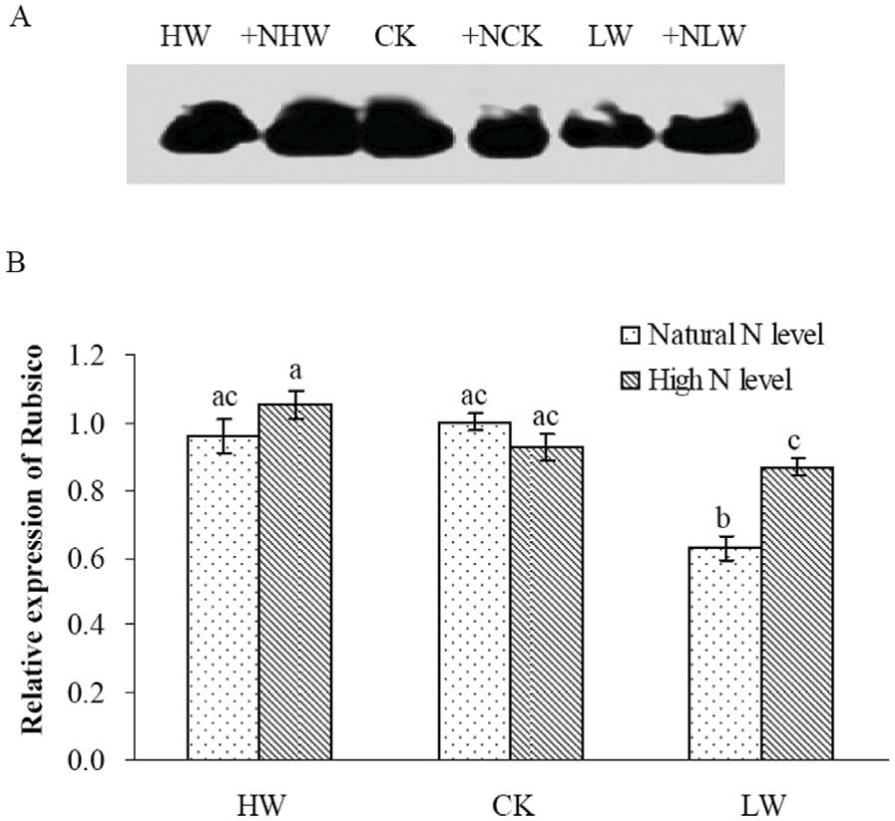
Western blot analysis of large subunits of Rubisco in leaves of *F. mandshurica* seedlings (panel A). The relative expression level is shown as the ratio of the band intensities between different treatments and CK with the analysis by Quantity One software (panel B).

In addition to the activity and activation state of Rubisco, soil N and water might affect photosynthetic responses by regulating expression of Rubisco in the leaves of the seedlings. Therefore, we further analyzed the effects of N addition and water regimes on the expression of the large subunits in Rubisco in the leaves of *F. mandshurica* seedlings. The leaf protein of *F. mandshurica* seedlings was separated by means of SDS-PAGE, and a 55 kDa distinct band showed up by western blot analysis (Fig. 7A). The analysis of band intensity indicated that the expression of Rubisco was down-regulated by LW treatment, while the expression of the protein wasn't significantly influenced in HW treatment (Fig. 7B). The expression level of Rubisco was higher in N addition and LW treatment than that in LW condition, suggesting that N addition increased the protein expression of Rubisco under LW condition. However, under HW and CK conditions, N addition didn't induce a change in the expression of.

### Immunolocalization of Rubisco

**Figure 8:**
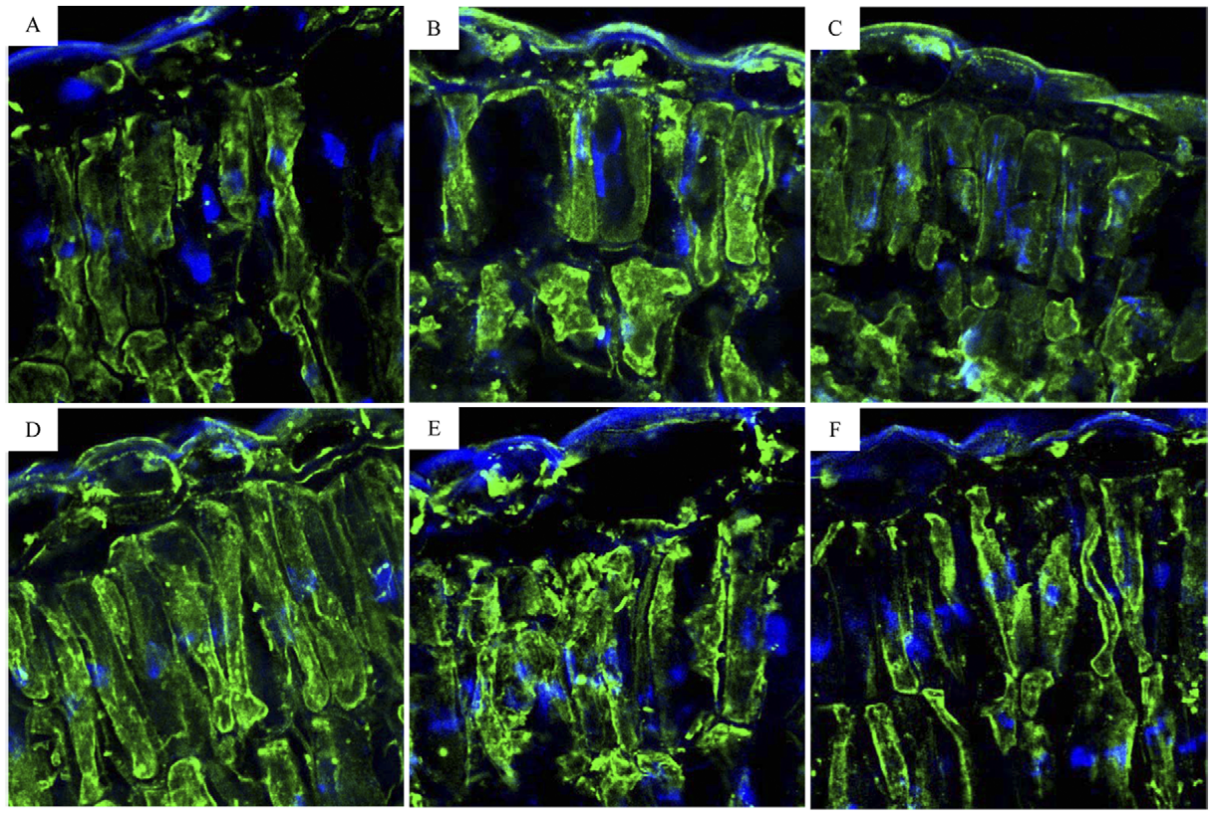
Confocal microscopy to show *in situ* immunolocalization of Rubisco in leaves of *F. mandshurica* seedlings. Label appears as green particles and nuclei are stained with DAPI (blue). (A) HW; (B) CK; (C) LW; (D) +NHW; (E) +NCK; (F) +NLW.

We further detected the distribution of Rubisco in the leaves of *F. mandshurica* seedlings. Immunofluorescent staining showed that Rubisco was found in chloroplasts throughout the leaf chlorenchyma in the seedlings (Fig. 8). Labeling for Rubisco was abundant in chloroplasts of leaf tissues of HW and CK, while the labeling was relatively low in leaves of the seedlings under LW condition. We observed that labeling for Rubisco was also concentrated in the chloroplasts of leaf chlorenchyma after N addition. The accumulation of Rubisco has increased in leaf tissues of LW by N addition, which was similar to the result from the expression of Rubisco by Western blots.

## Discussion

Soil N and water content are coupled tightly to the growth of plants. However, the interactive effects of N addition and soil water on plant physiological responses of tree seedlings have received relatively little attention [34]. In this study, we demonstrated significant interactive effects of N addition and soil water on the whole-plant growth and photosynthetic capacity of *F. mandschurica* seedlings in the temperate forest ecosystem in northeastern China. We showed that N addition increased seedling growth including plant height, total biomass and aboveground biomass under different soil water conditions. The reduction of the seedlings growth induced by low water supply was significantly attenuated by N addition. We observed the strong photosynthetic responses of *F. mandschurica* seedlings to N addition and water regimes. There were significant differences in leaf N content between soil water treatments, and an interactive effect of soil N and water on the leaf N content was found. Leaf N content displayed significant positive correlation with *A_max_* and also with SLA. N addition changed the photosynthetic capacity of seedlings under high water and natural conditions. N addition led to a significant increase in leaf chlorophyll content and the initial activity of Rubisco regardless of soil water condition. The protein expression of Rubisco was increased by N addition under LW condition. Immunofluorescent staining showed that the labeling for Rubisco was relatively low in leaves of the seedlings under LW condition. The accumulation of Rubisco was increased in leaf tissues of LW by N addition.

In the present study, we found the interactive effects of N addition and soil water on the whole-plant growth of *F. mandshurica* seedlings in the temperate forest ecosystem. N addition stimulated the growth of the seedlings under different soil water conditions, as reflecting by a significant increase in seedling growth parameters such as plant height, root collar diameter, total biomass and aboveground biomass (Table 1). Similar effects of N addition on plant growth have been reported for annual grass and wheat [46], [47]. The growth response of the seedlings to N addition and soil water suggested that N supply could amplify the positive effects of elevated soil moisture on plant growth [48]. In addition, the growth of the seedlings was negatively affected by LW treatment and this tendency was partially diminished by N addition, which was consistent with the previous finding in *Sophora davidii* seedlings [20]. These results indicated that N addition might alleviate the negative effects of LW manipulation on whole-plant growth of the seedlings. It demonstrated that N addition might play a key role in maintaining plant productivity under different soil water conditions in the temperate forest ecosystem.

The shifts in biomass allocation had an important impact on tree growth in the acclimation to changes of soil nutrient and water content [49], [50]. The ratio of stem and root biomass (S/R) was an indicator that represented demand-supply balance for environmental stresses [51]. Nutrient limitation and drought stress were found to increase carbon translocation from the leaves to the roots, thereby decreased the S/R ratio [52], [53]. Similar result was presented in our study, as the S/R ratio decreased with decreasing soil water content (Fig. 1F, *P*<0.05), which supported the assumption that reduced soil water content could lead to carbohydrate accumulation in the roots of plants [53]. Our results provided the evidence that N addition did not drive an alternation in the ratio of the aboveground and belowground biomass in seedlings. Biomass allocation for *F. mandshurica* seedlings might be not primarily N limited.

This study added important evidence in the interactive effects of N addition and soil water on the photosynthetic rate of *F. mandshurica* seedlings and the investigations conducted in the durum wheat [46] and hybridizing species [54]. We showed a strong interaction between N and soil water on *A_max_* of the seedlings in this ecosystem. N addition significantly enhanced the effect of HW regime on *A_ma_*_x_. The photosynthetic responses to N availability have been well documented in hardwood tree species [55]–[57], which indicated that the photosynthetic rate of the seedlings might be dependent on soil N availability in the temperate ecosystem. We further investigated the changes of SLA and leaf N content to explain the potential mechanism in leaf photosynthesis. SLA and leaf N content were both significantly correlated with *A_ma_*_x_ of the seedlings (Fig. 4). The results were similar to that found in boreal tree species and wheat [46], [58]. In addition, N addition triggered a significant increase in the stomatal diffusive conductance to H_2_O (*g_s_*) of the seedlings under HW condition (Fig. 2B). It is likely that N addition accelerate the transport of photosynthetic CO_2_ in the leaves, leading to enhanced *A_ma_*_x_ of the seedlings.

Leaf chlorophyll content is a good indicator of photosynthetic capacity. Low concentrations of chlorophyll limit photosynthetic potential directly and lead to a decrease in biomass production in the plants [59]. In this study, a strong interaction of soil N and water on leaf chlorophyll including total chlorophyll (a+b), Chl *a* and Chl *b* were found. Total chlorophyll (a+b), Chl *a* and Chl *b* content per unit area were all significantly increased in response to N addition in different soil water treatments (Fig. 5). The effects of N addition on chlorophyll were in agreement with the previous findings [20], [60], [61]. We also noticed a significant increase in Car/Chl ratio under LW condition.

Rubisco is a key enzyme in photosynthesis and its activity is the main limitation for photosynthetic CO_2_ fixation [62], [63]. Increased N availability may affect the photosynthesis of plants by altering the activity of Rubisco in the leaves [13], [64], [65]. Previous studies suggested that leaf *A_max_* is associated with Rubisco activity [66] or its activation state [67]. In our study, increased initial activity of Rubisco and its activation state were found in the leaves of the seedlings under N addition and different soil water conditions (Fig. 6). Total activity of Rubisco in the leaves also augmented with N addition under HW and CK conditions. A highly positive correlation was observed between the initial activity of Rubisco and leaf *A_max_* of the seedlings (data not shown). These results indicated that the initial activity of Rubisco was more closely involved in the regulation of the photosynthetic rate than total Rubisco activity or its activation state in *F. mandshurica* seedlings. The result was consistent with the previous findings in *Pinus pinaster* [68]. It was known that Rubisco activity increased linearly with leaf N in plants [69]–[72]. We observed that leaf N content was increased with N addition in the seedlings (Fig. 3), which might at least in part explained the increase of Rubisco activity.

It has been reported that the protein synthesis of Rubisco was influenced by leaf N content [73], [74], we therefore hypothesized that differences in photosynthetic response to N addition may be due to the expression of Rubisco in the leaves in *F. mandshurica* seedlings. In this study, the protein expression of Rubisco under N addition and water regimes were determined by immunoblotting and immuno-labeled techniques. We found that the expression of Rubisco in the leaves was down-regulated under LW condition and the tendency was reversed by N addition in the seedlings, indicating that N addition could alleviate the negative response of Rubisco to LW. A lower expression level of Rubisco and photosynthesis down-regulation were found in seedlings only in severe drought situation [75]. Immunofluorescent staining for Rubsico showed that the immunolocalization of Rubisco occurred in chloroplasts of the leaves in the seedlings. Low accumulation of Rubisco was detected in the leaves of the seedlings grown in LW condition, while labeling for Rubisco in the chloroplasts was increased by N addition in LW condition. These results were consistent with the findings from Western blots (Fig. 7, 8). The amount of Rubisco was usually considered to be much greater than required for photosynthesis under a wide range of environmental conditions [76]–[78]. Under plentiful soil water, N addition might not stimulate Rubisco expression and therefore no significant effect on leaf Rubisco content was showed by N addition. These results provided increasing evidence that Rubisco in the leaves might be in excess and function as an N store in normal environmental condition for *F. mandshurica* seedlings in the temperate forest ecosystem. The amount of Rubisco in the leaf was determined by the balance between its synthesis and degradation [79], [80]. With decreasing soil water content, the balance between Rubisco synthesis and degradation might be disrupted, and more Rubisco was degraded in response to water stress, which led to a decrease in the amount of leaf Rubisco. N addition might significantly increase the expression of Rubisco in the leaves to alleviate the negative response of photosynthesis in the seedlings. Therefore, in addition to effects of the enzyme activity and activation state, N addition might affect the photosynthetic rate of the seedlings by regulating the expression of Rubisco in the leaves under low soil moisture in the temperate forest ecosystem.

In summary, this study evaluated for the first time the interactive effects of N and soil water on the growth and photosynthetic responses of *F. mandschurica* seedlings in the temperate ecosystem in northeastern China. We demonstrated that the growth of the seedlings was positively affected by combined manipulations of N addition and soil water. N addition significantly enhanced the growth and biomass production of the seedlings under plentiful soil water condition and could alleviate the negative effect of LW treatment on plant growth. Furthermore, N addition could lead to a dramatic increase in the photosynthetic capacity under high-water and natural conditions, which was paralleled with the shifts of leaf chlorophyll content and Rubisco enzymatic activity. Rubisco expression was up-regulated by N addition in LW condition, which might be implicated in maintaining the balance of its synthesis and degradation. Our data provided increasing evidence that N deposition might be beneficial to biomass production and photosynthesis in forest seedlings in the temperate ecosystem.

## References

[1] Matson P, Lohse KA, Hall SJ (2002). The globalization of nitrogen addition: consequences for terrestrial ecosystems.. Ambio.

[2] Aber JD, Goodale CL, Ollinger SV, Smith ML, Magill AH (2003). Is nitrogen deposition altering the nitrogen status of northeastern forests?. BioScience.

[3] Compton JE, Watrud LS, Porteous LA, DeGrood S (2004). Response of soil microbial biomass and community composition to chronic nitrogen additions at Harvard forest.. Forest Ecology and Management.

[4] Schwinning S, Starr BI, Wojcik NJ, Miller ME, Ehleringer JE (2005). Effects of nitrogen deposition on arid grassland in the Colorado Plateau cold desert.. Rangeland Ecology & Management.

[5] Vitousek PM, Howarth RW (1991). Nitrogen limitation on land and in the sea: how can it occur.. Biogeochemistry.

[6] LeBauer DS, Treseder KK (2008). Nitrogen limitation of net primary productivity in terrestrial ecosystems is globally distributed.. Ecology.

[7] Catovsky S, Bazzaz FA (2002). Nitrogen availability influences regeneration of coniferous and broad-leaved tree species in the understory seedling bank.. Ecological Applications.

[8] Correia CM, Moutinho Pereira JM, Coutinho JF, Björn LO, Torres-Pereira JMG (2005). Ultraviolet-B radiation and nitrogen affect the photosynthesis of maize: a Mediterranean field study.. Eur J Agron.

[9] Latham RE (1992). Co-occurring tree species change rank in seedling performance with resources varied experimentally.. Ecology.

[10] Mitchell A, Hinckley T (1993). Effects of foliar nitrogen concentration on photosynthesis and water used efficiency in Douglas-fir.. Tree Physiol.

[11] Ripullone F, Grassi G, Lauteri M, Amato M, Borghetti M (2003). Photosynthesis–nitrogen relationships: interpretation of different patterns between Pseudotsuga menziesii and Populus euroamericana in a ministand experiment.. Tree Physiol.

[12] Vaitkus MR, Ciravolo TG, McLeod KW, Mavity EM, Novak KL (1993). Growth and photosynthesis of seedlings of five bottomland tree species following nutrient enrichment.. Am Midl Nat.

[13] Field C, Mooney HA, Givnish T The photosynthesis–nitrogen relationship in wild plants.. On the economy of plant form and function.

[14] Egli P, Schmid B (1999). Relationships between leaf nitrogen and limitations of photosynthesis in canopies of Solidago altissima.. Acta Oecol.

[15] Shangguan ZP, Shao MA, Dyckmans J (2000). Nitrogen nutrition and water stress effects on leaf photosynthetic gas exchange and water use efficiency in winter wheat.. Environ Exp Bot.

[16] DaMatta FM, Loos RA, Silva EA, Loureiro ME, Ducatti C (2002). Effects of soil water deficit and nitrogen nutrition on water relations and photosynthesis of pot-grown Coffea canephora Pierre.. Trees.

[17] Chandler JW, Dale JE (1995). Nitrogen deficiency and fertilization effects on needle growth and photosynthesis in Sitka spruce (Picea sitchensis).. Tree Physiology.

[18] Lauter DJ, Munns DN, Clarkin KL (1981). Salt response of chickpea as influenced by N deposition.. Agron J.

[19] Yin CY, Berninger F, Li CY (2006). Photosynthetic responses of Populus przewalski subjected to drought stress.. Photosynthetica.

[20] Wu FZ, Bao WK, Li LF, Wu N (2008). Effects of water stress and nitrogen deposition on leaf gas exchange and fluorescence parameters of Sophora davidii seedlings.. Photosynthetica.

[21] Penuelas J, Rutishauser T, Filella I (2009). Phenology feedbacks on climate change.. Science.

[22] Patrick LD, Ogle K, Tissue DT (2009). A hierarchical Bayesian approach for estimation of photosynthetic parameters of C_3_ plants.. Plant, Cell Environ.

[23] Lawlor DW (1995). Photosynthesis, productivity and environment.. J Exp Bot.

[24] Brestic M, Cornic G, Fryer MJ, Baker NR (1995). Does photorespiration protect the photosynthetic apparatus in Frech bean leaves from photoinhibition during drought stree?. Planta.

[25] Foyer CH, Valadier MH, Migge A, Becker TW (1998). Drought-induced effects on nitrate reductase activity and mRNA and on the coordination of nitrogen and carbon metabolism in maize leaves.. Plant Physiology.

[26] Chaves MM, Oliveira MM (2004). Mechanisms underlying plant resilience to water deficits: prospects for water-saving agriculture.. Journal of Experimental Botany.

[27] Flexas J, Bota J, Loreto F, Cornic G, Sharkey TD (2004). Diffusive and metabolic limitations to photosynthesis under drought and salinity in C3 plants.. Plant Biology.

[28] Ennahli S, Earl HJ (2005). Physiological limitations to photosynthetic carbon assimilation in cotton under water stress.. Crop Science.

[29] Grassi G, Magnani F (2005). Stomatal, mesophyll conductance and biochemical limitations to photosynthesis as affected by drought and leaf ontogeny in ash and oak trees.. Plant, Cell & Environment.

[30] Alves AAC, Setter TL (2000). Response of Cassava to Water Deficit: Leaf Area Growth and Abscisic Acid.. Crop Sci.

[31] Huang Y, Rothwell JC, Edwards MJ, Chen RS (2008). Effect of Physiological Activity on an NMDA-Dependent Form of Cortical Plasticity in Human.. Cereb Cortex.

[32] Liu P, Huang J, Han X, Sun OJ, Zhou Z (2006). Differential responses of litter decomposition to increased soil nutrients and water between two contrasting grassland plant species of Inner Mongolia, China.. Applied Soil Ecology.

[33] Wang C, Wan S, Xing X, Zhang L, Han X (2006). Temperature and soil moisture interactively affected soil net N mineralization in temperate grassland in Northern China.. Soil Biology and Biochemistry.

[34] McDonald AJS, Davies WJ (1996). Keeping in touch: responses of the whole plant to deficits in water and nitrogen supply.. Advances in Botanical Research.

[35] Andersson I, Backlund A (2008). Structure and function of Rubisco.. Plant PhysioI Biochem.

[36] Warren CR, Dreyer E, Adams MA (2003). Photosynthesis-Rubisco relationships in foliage of Pinus sylvestris in response to nitrogen supply and the proposed role of Rubisco and amino acids as nitrogen stores.. Trees.

[37] Gezelius K (1986). Free amino acids and nitrogen during shoot development in Scats pine seedlings.. Physiol Plant.

[38] Laitinen K, Luomala E-M, Kellom ki S, Vapaavuori E (2000). Carbon assimilation and nitrogen in needles of fertilized and unfertilized field-grown Scots pine at natural and elevated concentrations of CO_2_.. Tree Physiol.

[39] Zhang M, Guan DX, Han SJ, Wu JB, Zhang JH (2005). Climatic dynamics of broadleaved Korean pine forest in Changbai Mountain during the last 22 years.. Chinese Journal of Ecology.

[40] Ellsworth DS (2000). Seasonal CO_2_ assimilation and stomatal limitations in a Pinus taeda canopy.. Tree Physiology.

[41] Yamaguchi, Masashi (2007). Pneumatic tire..

[42] Inskeep WP, Bloom PR (1985). Extinction Coefficients of Chlorophyll a and b in *N,N*-Dimethylformamide and 80% Acetone.. Plant Physiol.

[43] Arnon DI (1949). Copper enzymes in isolated chloroplasts. Polyphenoloxidae in Beta vulgaris.. Plant Physiol.

[44] Tissue DT, Thomas RB, Strain BR (1993). Long-term effects of elevated CO_2_ and nutrients on photosynthesis and Rubisco in loblolly pine seedlings.. Plant Cell and Environment.

[45] Bradford MM (1976). Anal Biochem..

[46] Cabrera-Bosquet L, Molero G, Bort J, Nogues S, Araus JL (2007). The combined effect of constantwater deficit and nitrogen supply on WUE, NUE and Delta C-13 in durum wheat potted plants.. Ann Appl Biol.

[47] Zhou X, Zhang Y, Ji X, Alison D, Serped M (2011). Combined effects of nitrogen deposition and water stress on growth and physiological responses of two annual desert plants in northwestern China.. Environmental and Experimental Botany.

[48] Reich PB, Hungate BA, Luo YQ (2006). Carbon-nitrogen interactions in terrestrial ecosystems in response to rising atmospheric carbon dioxide.. Annu Rev Ecol Evol Syst.

[49] Poorter H, Remkes C (1990). Leaf area ratio and net assimilation rate of 24 wild species differing in relative growth rate.. Oecologia.

[50] Reich PB, Kloeppel BD, Ellsworth DS, Waiters MB (1995). Different photosynthesis-nitrogen relations in deciduous hardwood and evergreen coniferous tree species.. Oecologia.

[51] Lambers H, Chapin FS, Pons TL (1998). Plant Physiological Ecology..

[52] Andrews M, Sprent JI, Raven JA, Eady PE (1999). Relationships between shoot to root ratio, growth and leaf soluble protein concentration of Pisum sativum, Phaseolus vulgaris and Triticum aestivum under different nutrient deficiencies.. Plant Cell Environ.

[53] Poorter H, Nagel O (1999). The role of biomass allocation in the growth response of plants to different levels of light, CO_2_, nutrients and water: a quantitative review.. Aust J Plant Physiol.

[54] Campbell DR, Wu CA, Travers SE (2010). Photosynthetic and growth responses of reciprocal hybrids to variation in water and nitrogen availability.. American Journal of Botany.

[55] Wendler R, Millard P (1996). Impacts of water and nitrogen supplies on the physiology, leaf demography and nitrogen dynamics of Betula pendula.. Tree Physiol.

[56] Wang YP, Leuning R (1998). A two-leaf model for canopy conductance, photosynthesis and partitioning of available energy I: Model description and comparison with a multi-layered model.. Agricultural and Forest Meteorology.

[57] Tyree MC, Seiler JR, Maier CA (2009). Short-term impacts of nutrient manipulations on leaf gas exchange and biomass partitioning in contrasting 2-year-old Pinus taeda clones during seedling establishment.. Forest Ecology and Management.

[58] Reich PB, Walters MB, Tjoelker MG, Vanderklein DW, Buschena C (1998). Photosynthesis and respiration rates depend on leaf and root morphology and nitrogen concentration in nine boreal tree species differing in relative growth rate.. Functional Ecology.

[59] Naumann JC, Young DR, Anderson JE (2008). Leaf chlorophyll fluorescence, reflectance, and physiological response to freshwater and saltwater flooding in the evergreen shrub, Myrica cerifera.. Environmental and Experimental Botany.

[60] Van den Berg AK, Perkins TD (2004). Evaluation of portable chlorophyll meter to estimate chlorophyll and nitrogen contents in sugar maple (Acer saccharum Marsh.) leaves.. Forest Ecology and Management.

[61] Shaw B, Thomas TH, Cooke DT (2002). Responses of sugar beet (Beta vulgaris L.) to drought and nutrient deficiency stress.. Plant Growth Regul.

[62] Evans JR (1986). The relationship between carbon-dioxide-limited photosynthetic rate and ribulose-1,5-bisphosphate-carboxylase content in two nuclear-cytouplasm substitution lines of wheat, and the co-ordination of ribulose-bisphosphate-carboxylation and electron-transport capacities.. Planta.

[63] Makino A, Mae T, Ohira K (1984). Relation between nitrogen and Ribulose-1, 5-bisphosphate carboxylase in rice leaves from emergence through senescence.. Plant and Cell Physiology.

[64] Kutik J, Lubomir N, Demmers-Derks HH (1995). Chloroplast ultrastructure of sugar beet (Beta vulgaris L.) cultivated in normal and elevated CO_2_ concentrations with two contrasted nitrogen supplies.. Journal of Experimental Botany.

[65] Bondada BR, Syvertsen JP (2003). Leaf chlorophyll, net gas exchange and chloroplast ultrastructure in citrus leaves of different nitrogen status.. Tree Physiology.

[66] Bota J, Medrano H, Flexas J (2004). Is photosynthesis limited by decreased Rubisco activity and RuBP content under progressive water stress?. The New Phytologist.

[67] Manter DK, Kavanagh KL, Rose CL (2005). Growth response of Douglas-fir seedlings to nitrogen fertilization: importance of Rubisco activation state and respiration rates.. Tree Physiology.

[68] Warren CR, Adams MA (2001). Distribution of N, Rubisco and photosynthesis in Pinus pinaster and acclimation to light.. Plant Cell Environ.

[69] Evans JR (1983). Nitrogen and photosynthesis in the flag leaf of wheat (Triticum aestivum L).. Plant physiology.

[70] Sage RF, Pearcy RW, Seemann JR (1987). The nitrogen use efficiency of C3 and C4 plants. III. Leaf nitrogen effects on the activity of carboxylating enzymes in Chenopodium album (L.) and Amaranthus retroflexus (L.).. Plant Physiology.

[71] Nakano H, Makino A, Mae T (1997). The effect of elevated partial pressure of CO_2_ on the relationship between photosynthetic capacity and N content in rice leaves.. Plant Physiology.

[72] Cheng L, Fuchigami LH (2000). CO_2_ assimilation in relation to nitrogen in apple leaves.. Journal of Horticultural Science and Biotechnology.

[73] Stitt M, Baker NR (1996). Metabolic regulation of photosynthesis.. Photosynthesis and the Environment.

[74] Nakaji T, Fukami M, Dokiya Y, Izuta T (2001). Effects of high nitrogen load on growth, photosynthesis and nutrient status of Cryptomeria japonica and Pinus densiflora seedlings.. Trees.

[75] Asghari R, Ebrahimzadeh H (2006). Drought stress increases the expression of wheat leaf Ribulose 1,5-Bisphosphate carboxylase/oxyenase protein.. Iranian Journal of Science and Technology, Transaction.

[76] Stitt M, Schulze ED (1994). Does Rubisco control the rate of photosynthesis and plant growth? An exercise in molecular ecophysiology.. Plant Cell Environ.

[77] Eichelmann H, Laisk A (1999). Ribulose-1, 5-bisphosphate carboxylase/oxygenase content, assimilatory charge, and mesophyll conductance in leaves.. Plant Physiol.

[78] Warren CR, Adams MA (2000). Capillary electrophoresis for the determination of major amino acids and sugars in foliage: application to the nitrogen nutrition of sclerophyllous species.. J Exp Bot.

[79] Mae T, Makino A, Ohira K (1983). Changes in the amounts of Ribulose bisphosphate carboxylase synthesized and degraded during the life-span of rice leaf (Oryza sativa L).. Plant and Cell Physiology.

[80] Makino A, Mae T, Ohira K (1988). Differences between wheat and rice in the enzymic properties of ribulose-l,5-bisphosphate carboxylase/oxygenase and the relationship to photosynthetic gas exchange.. Planta.

